# Supplementary material for: Checkpoint Kinase 2 Inhibition Can Reverse Tamoxifen Resistance in ER-Positive Breast Cancer
Source: Int J Mol Sci. 2022 Oct 14;23(20):12290. doi: 10.3390/ijms232012290 (PMC9604393; doi:10.3390/ijms232012290)
Supplement: Supplementary file 1 [file ijms-23-12290-s001.zip › ijms-1930677-supplementary.pdf]

Figure S1

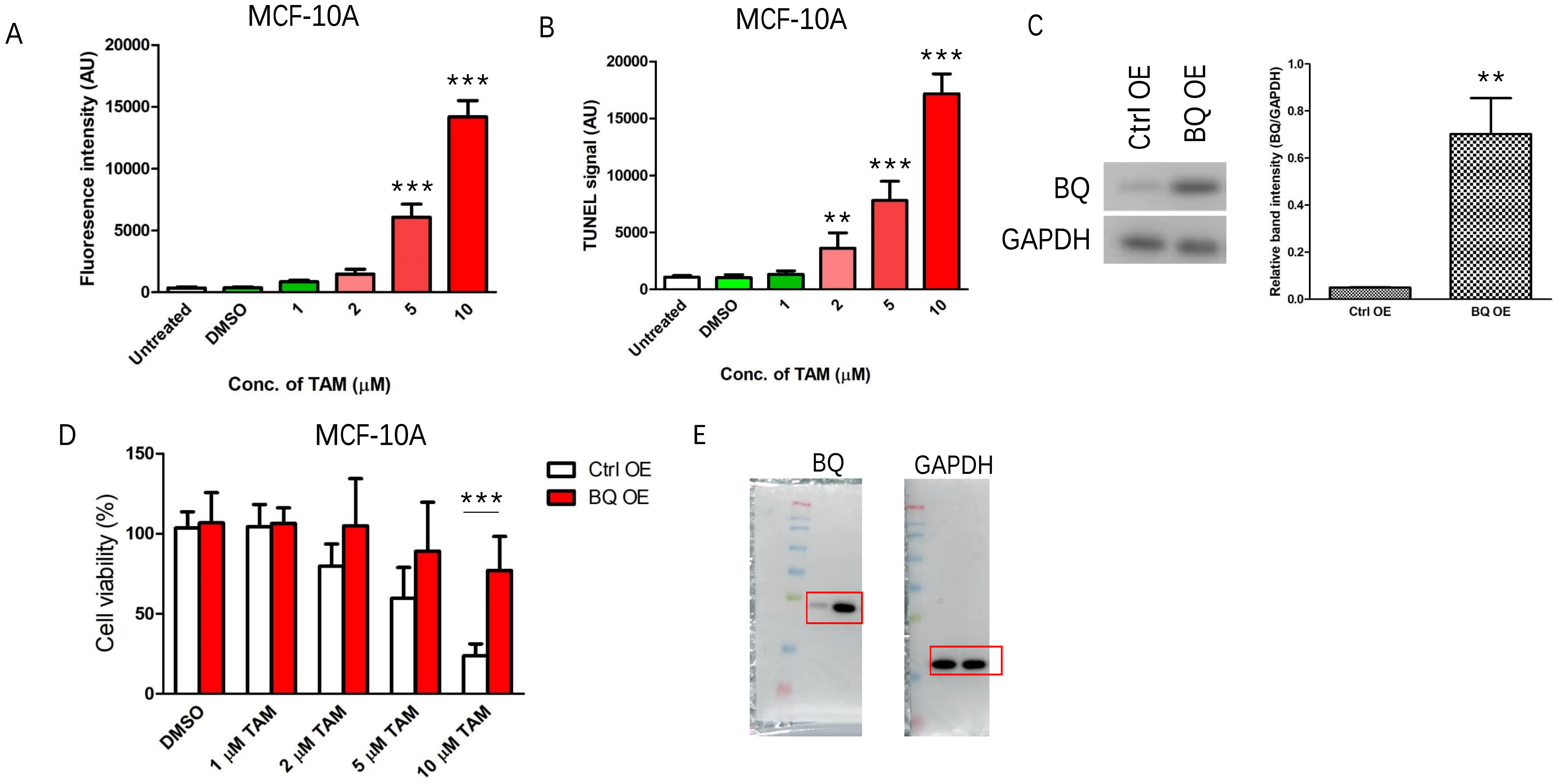

A
MCF-10A
Fluoresence intensity (AU)
20000
15000
10000
5000
0
***
***
Untreated
DMSO
1
2
5
10
Conc. of TAM (μM)
B
MCF-10A
TUNEL signal (AU)
20000
15000
10000
5000
0
**
***
***
Untreated
DMSO
1
2
5
10
Conc. of TAM (μM)
C
Ctrl OE
BQ OE
BQ
GAPDH
Relative band intensity (BQ/GAPDH)
1.0
0.8
0.6
0.4
0.2
0.0
**
Ctrl OE
BQ OE
D
MCF-10A
Cell viability (%)
150
100
50
0
***
Ctrl OE
BQ OE
DMSO
1 μM TAM
2 μM TAM
5 μM TAM
10 μM TAM
E
BQ
GAPDH

Figure S1 Overexpression of BQ could compromise the effect of tamoxifen (TAM) in non-cancerous breast cell MCF-10A. A) The effect of TAM on ROS formation in MCF-10A. The cells were treated with different concentrations of TAM for 72 hours. ROS was determined by CM-H_2DCFDA. The fluorescence signal represents ROS level. B) The effect of TAM on DNA damage. TUNEL was employed to detect DNA damage. The cells were treated with different concentrations of TAM for 72 hours. Results were shown as mean ± SD from six independent experiments. One-way ANOVA with Bonferroni's post-test was used to compare the statistical significance with the untreated group. C) Overexpression of BQ in MCF-10A. The cells were transfected with pcDNA3.1-His-BQ323636.1. Proteins were harvested 48 hours post-transfection. Western blot with anti-BQ antibody was used to detect BQ expression. D) Overexpression of BQ could compromise the effect of TAM on MCF-10A. The cells were treated with TAM for 96 hours. MTT assay was used to determine cell viability. Results were shown as mean ± SD from six independent experiments. Two-way ANOVA with Bonferroni's post-test was used to compare the statistical significance between the indicated groups. E) Representative raw blots in Figure S1C. ** and *** represent $p < 0.01$ and $p < 0.001$, respectively.

Figure S2

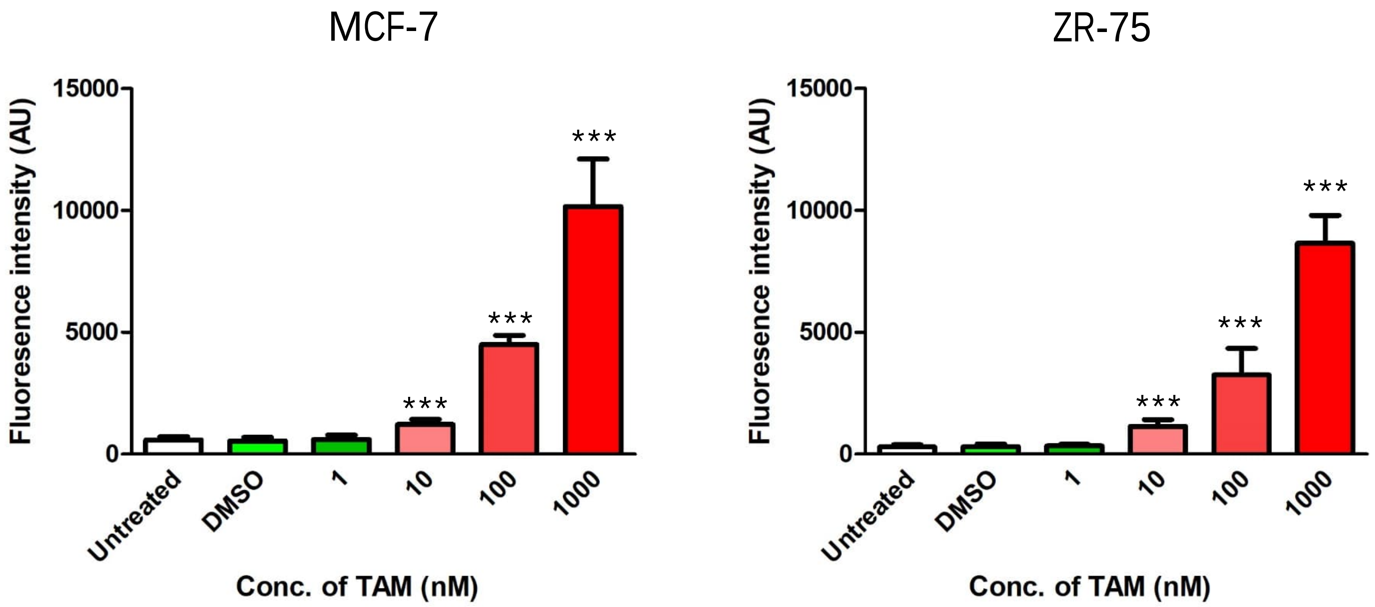
MCF-7
ZR-75
Fluoresence intensity (AU)
15000
10000
5000
0
***
Untreated
DMSO
1
10
100
1000
Conc. of TAM (nM)

Figure S2 The effect of TAM on ROS formation in MCF-7 and ZR-75. The cells were treated with TAM for 72 hours. ROS was determined by CM-H_2DCFDA. The fluorescence signal represents ROS level. Results were shown as mean ± SD from six independent experiments. One-way ANOVA with Bonferroni's post-test was employed to compare the statistical significance with untreated group. *** represents $p < 0.001$,

Figure S3

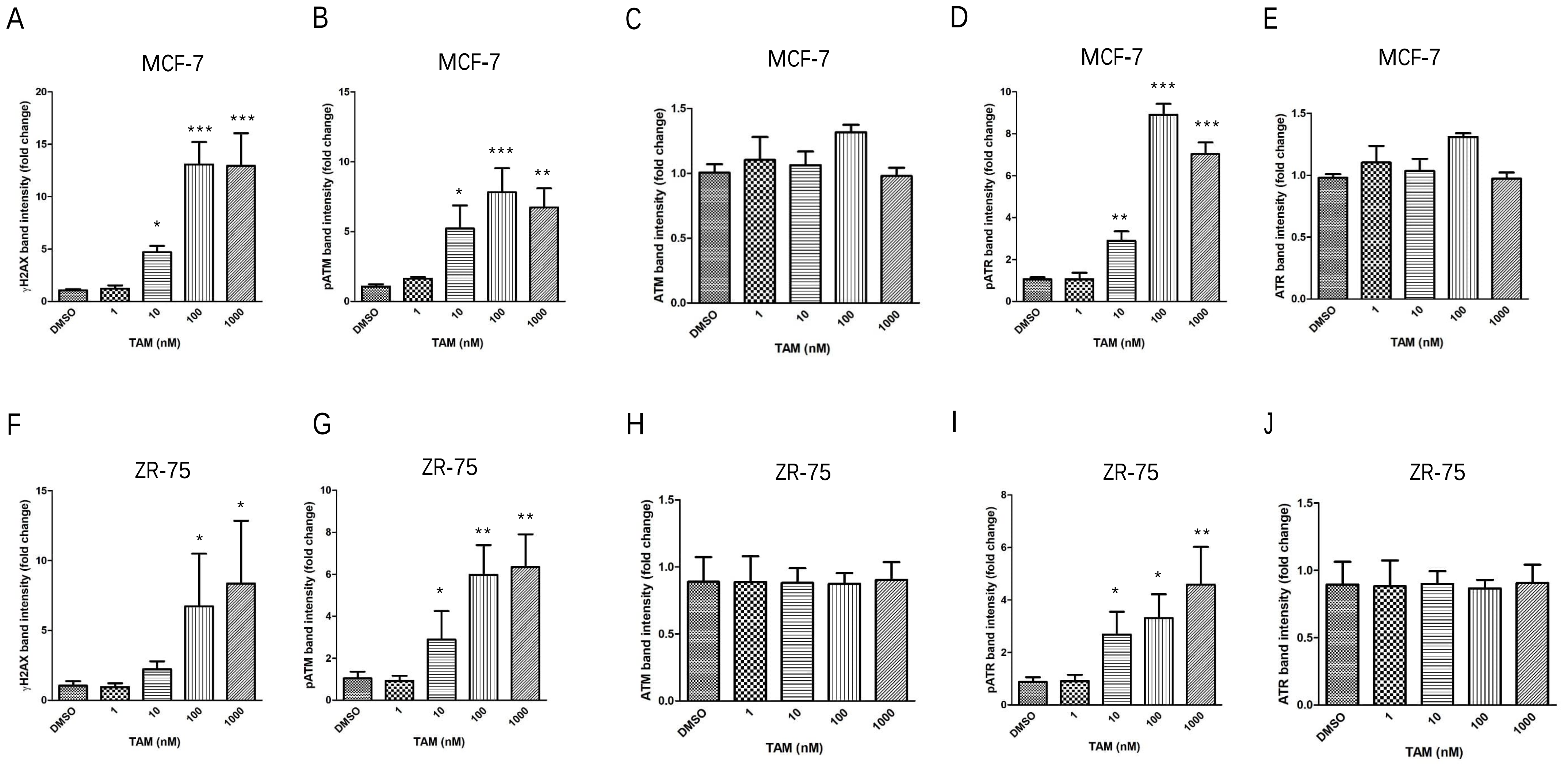
A
MCF-7
γH2AX band intensity (fold change)
TAM (nM)
DMSO
1
10
100
1000
B
MCF-7
pATM band intensity (fold change)
TAM (nM)
C
MCF-7
ATM band intensity (fold change)
TAM (nM)
D
MCF-7
pATR band intensity (fold change)
TAM (nM)
E
MCF-7
ATR band intensity (fold change)
TAM (nM)
F
ZR-75
γH2AX band intensity (fold change)
TAM (nM)
G
ZR-75
pATM band intensity (fold change)
TAM (nM)
H
ZR-75
ATM band intensity (fold change)
TAM (nM)
I
ZR-75
pATR band intensity (fold change)
TAM (nM)
J
ZR-75
ATR band intensity (fold change)
TAM (nM)

Figure S3

K

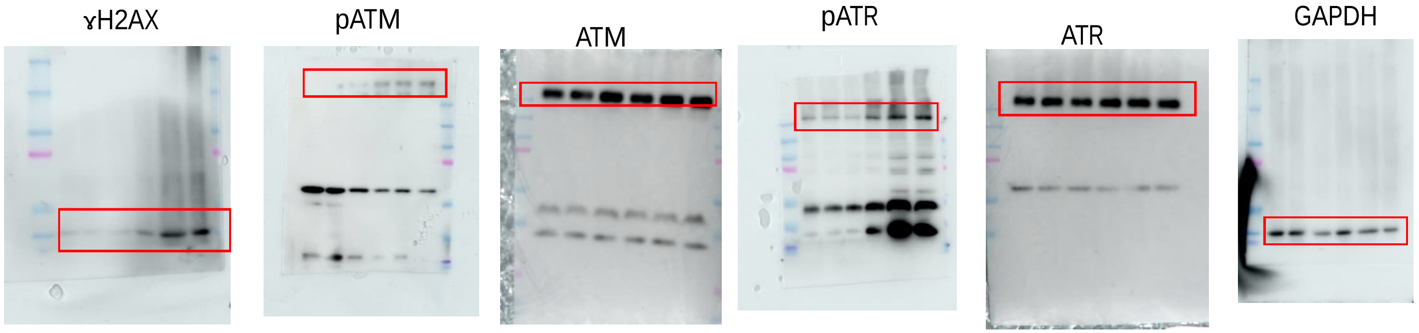

L

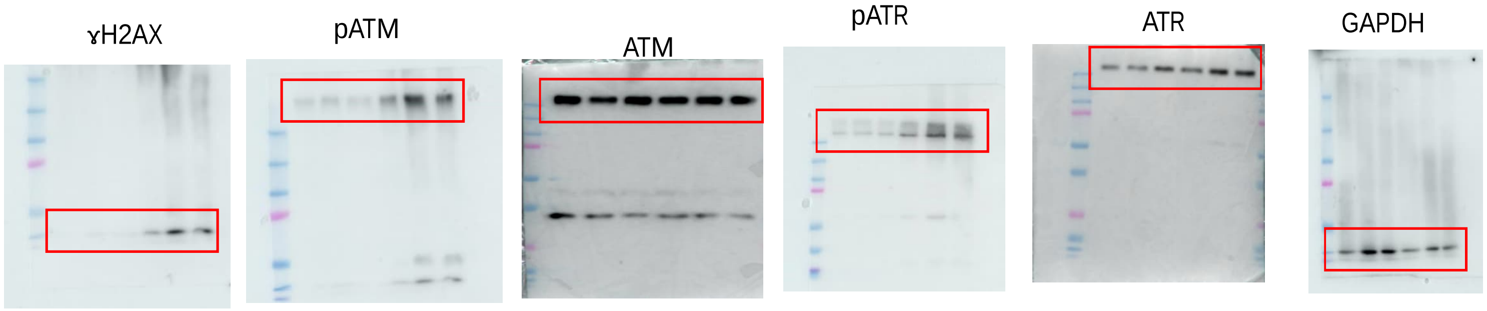

Figure S3 Quantification of A) ɤH2AX, B) p-ATM (Ser1981), C) ATM, D) p-ATR (Ser1989) and E) ATR band intensity of MCF-7 panel in Figure 1C. Quantification of F) ɤH2AX, G) p-ATM, H) ATM, I) p-ATR and J) ATR band intensity of ZR-75 panel in Figure 1C. Fold change to untreated control was determined. Results were shown as mean ± SD from three independent experiments. One-way ANOVA with Bonferroni's post-test was employed to compare the statistical significance with DMSO. *, **, and *** represent $p < 0.05$, $p < 0.01$, and $p < 0.001$, respectively. K) Representative raw blots for the MCF-7 panel in Figure 1C. L) Representative raw blots for the ZR-75 panel in Figure 1C.

Figure S4

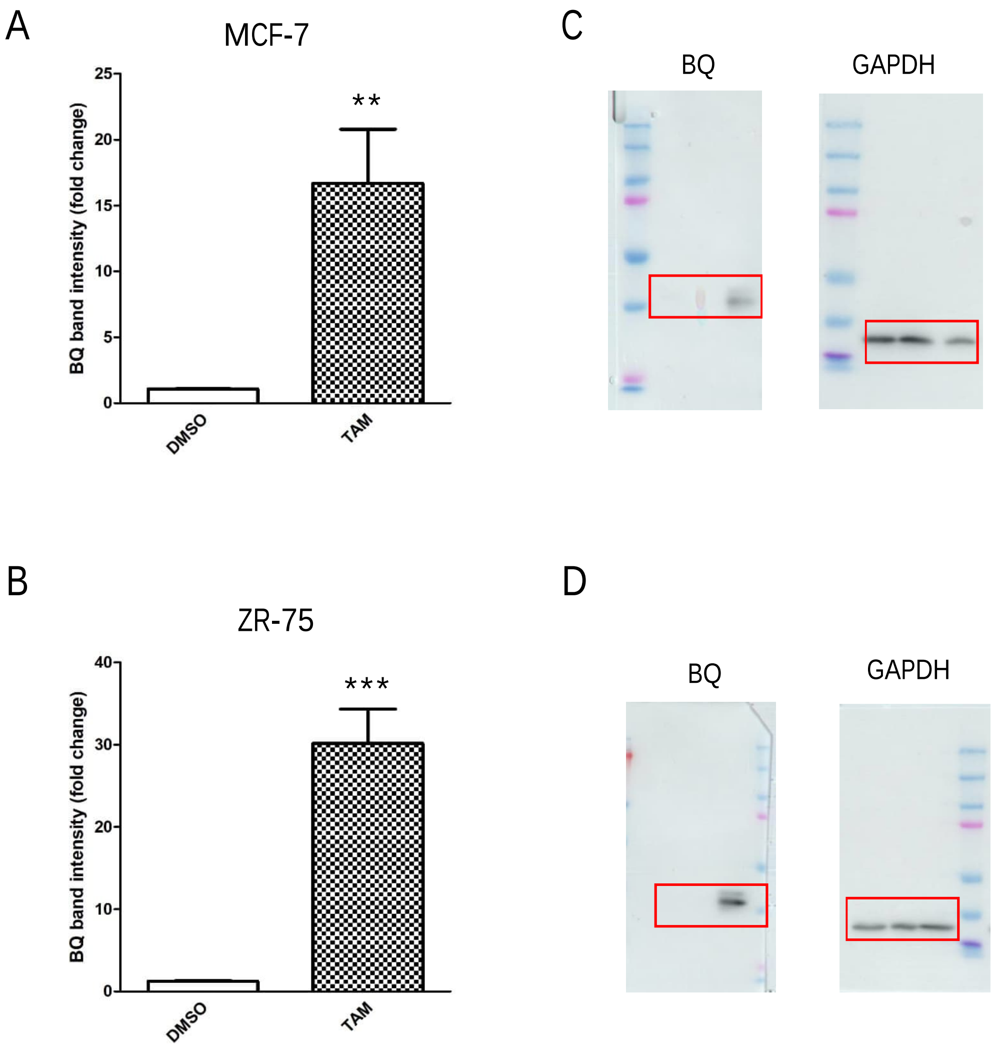

A
MCF-7
BQ band intensity (fold change)
25
20
15
10
5
0
**
DMSO
TAM
C
BQ
GAPDH
B
ZR-75
BQ band intensity (fold change)
40
30
20
10
0
***
DMSO
TAM
D
BQ
GAPDH

Figure S4 Quantification of BQ in A) MCF-7 and B) ZR-75 panel in Figure 1D. Results were shown as mean ± SD from three independent experiments. Students' t-test was employed to compare the statistical significance between groups. ** and *** represent $p < 0.01$, and $p < 0.001$, respectively. C) Representative raw blots for the MCF-7 panel in Figure 1D. D) Representative raw blots for the ZR-75 panel in Figure 1D.

Figure S5

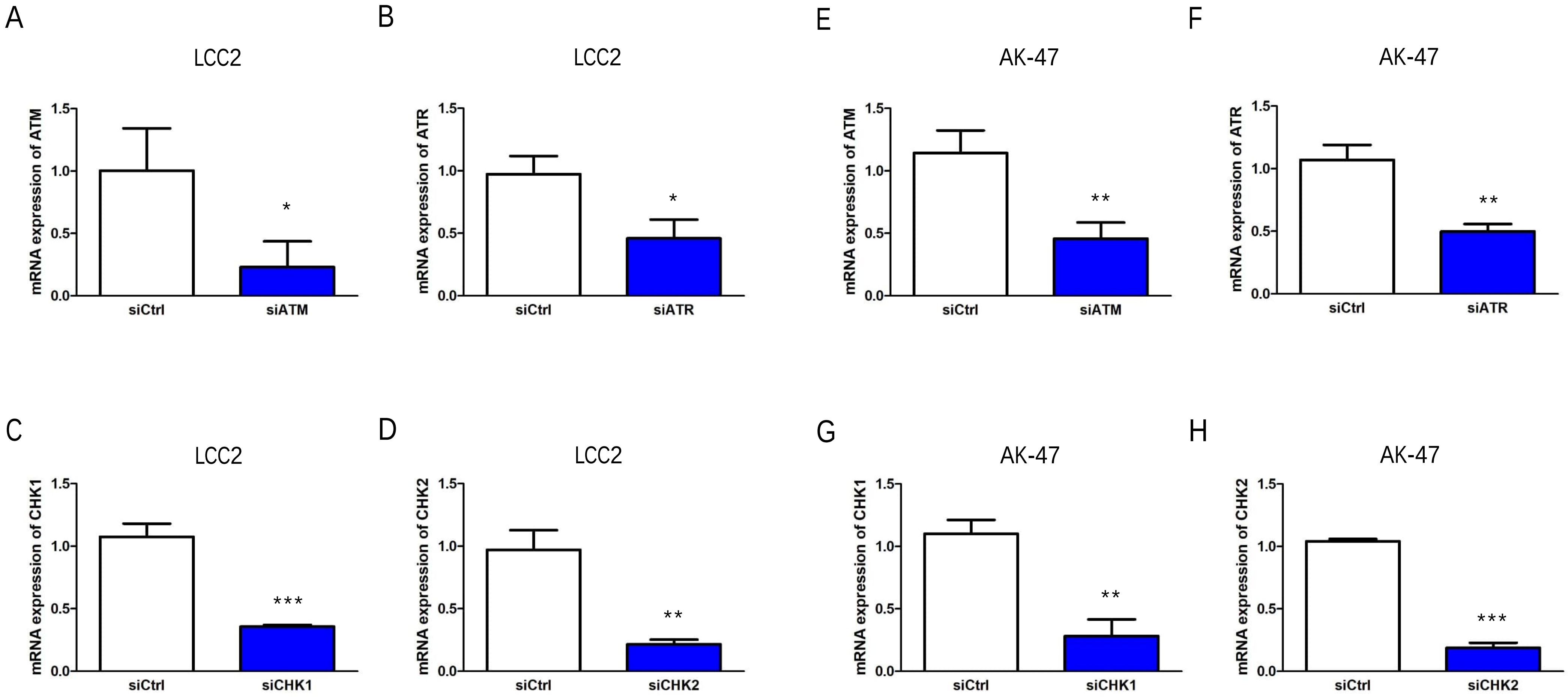
A
LCC2
mRNA expression of ATM
1.5
1.0
0.5
0.0
*
siCtrl
siATM
B
LCC2
mRNA expression of ATR
*
siCtrl
siATR
E
AK-47
mRNA expression of ATM
**
siCtrl
siATM
F
AK-47
mRNA expression of ATR
**
siCtrl
siATR
C
LCC2
mRNA expression of CHK1
***
siCtrl
siCHK1
D
LCC2
mRNA expression of CHK2
**
siCtrl
siCHK2
G
AK-47
mRNA expression of CHK1
**
siCtrl
siCHK1
H
AK-47
mRNA expression of CHK2
***
siCtrl
siCHK2

Figure S5 Knockdown efficiency of A) siATM, B) siATR, C) siCHK1, and D) siCHK2 in LCC2. Knockdown efficiency of E) siATM, F) siATR, G) siCHK1, and H) siCHK2 in AK-47. The cells were transfected with 20 nM of the siRNA for 72 hours. qPCR was employed to determine the relative gene expression level. GAPDH was used as the internal control. Results were shown as mean ± SD from three independent experiments. Students' t-test was employed to compare the statistical significance between groups. *, **, and *** represent $p < 0.05$, $p < 0.01$, and $p < 0.001$, respectively.

Figure S6

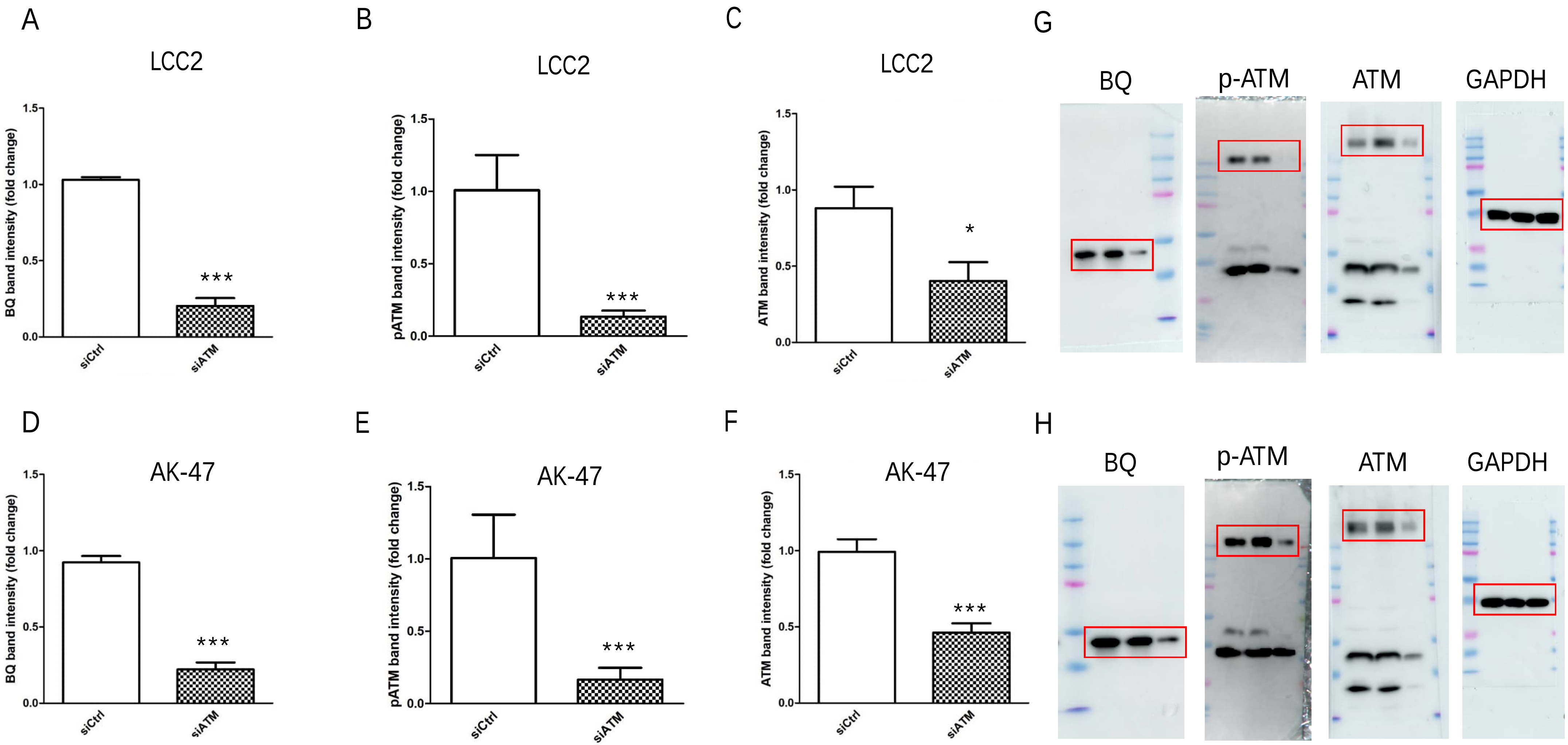
A
LCC2
BQ band intensity (fold change)
1.5
1.0
0.5
0.0
***
siCtrl
siATM
B
LCC2
pATM band intensity (fold change)
***
siCtrl
siATM
C
LCC2
ATM band intensity (fold change)
*
siCtrl
siATM
G
BQ
p-ATM
ATM
GAPDH
D
AK-47
BQ band intensity (fold change)
***
siCtrl
siATM
E
AK-47
pATM band intensity (fold change)
***
siCtrl
siATM
F
AK-47
ATM band intensity (fold change)
***
siCtrl
siATM
H
BQ
p-ATM
ATM
GAPDH

Figure S6 Quantification of A) BQ, B) p-ATM (Ser1981) and C) ATM band intensity of the LCC2 panel in Figure 2A. Quantification of D) BQ, E) p-ATM (Ser1981) and F) ATM band intensity of the AK-47 panel in Figure 2A. G) Representative raw blots for the LCC2 panel in Figure 2A. H) Representative raw blots for the AK-47 panel in Figure 2A. Results were shown as mean ± SD from three independent experiments. Students' t-test was employed to compare the statistical significance between groups. * and *** represent $p < 0.05$, and $p < 0.001$, respectively.

Figure S7

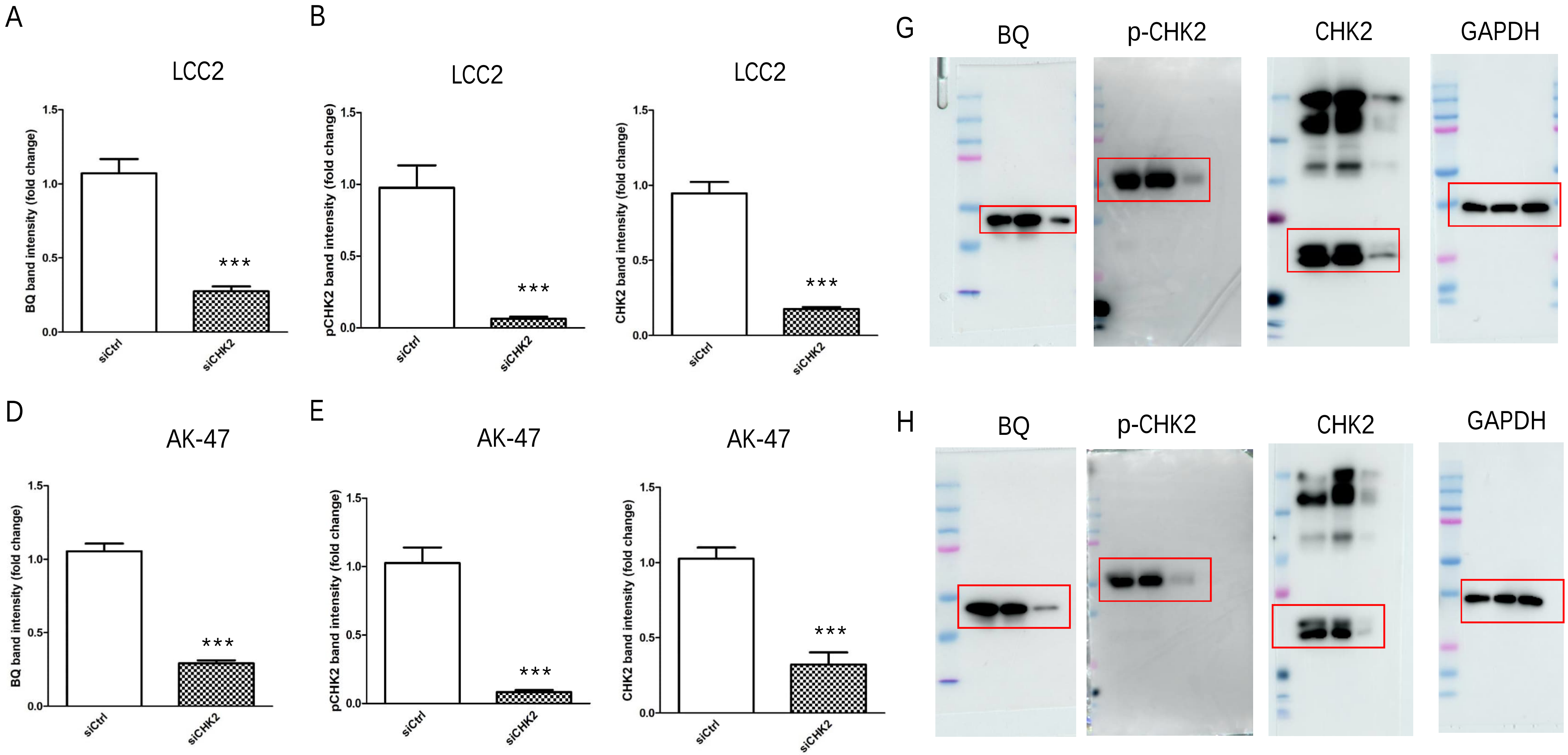
A
LCC2
BQ band intensity (fold change)
1.5
1.0
0.5
0.0
***
siCtrl
siCHK2
B
LCC2
pCHK2 band intensity (fold change)
LCC2
CHK2 band intensity (fold change)
D
AK-47
BQ band intensity (fold change)
E
AK-47
pCHK2 band intensity (fold change)
AK-47
CHK2 band intensity (fold change)
G
BQ
p-CHK2
CHK2
GAPDH
H
BQ
p-CHK2
CHK2
GAPDH

Figure S7 Quantification of A) BQ, B) p-CHK2 (Thr68) and C) CHK2 band intensity of the LCC2 panel in Figure 2B. Quantification of D) BQ, E) p-CHK2 (Thr68) and F) CHK2 band intensity of the AK-47 panel in Figure 2B. G) Representative raw blots for the LCC2 panel in Figure 2B. H) Representative raw blots for the AK-47 panel in Figure 2B. Results were shown as mean ± SD from three independent experiments. Students' t-test was employed to compare the statistical significance between groups. *** represents $p < 0.001$.

Figure S8

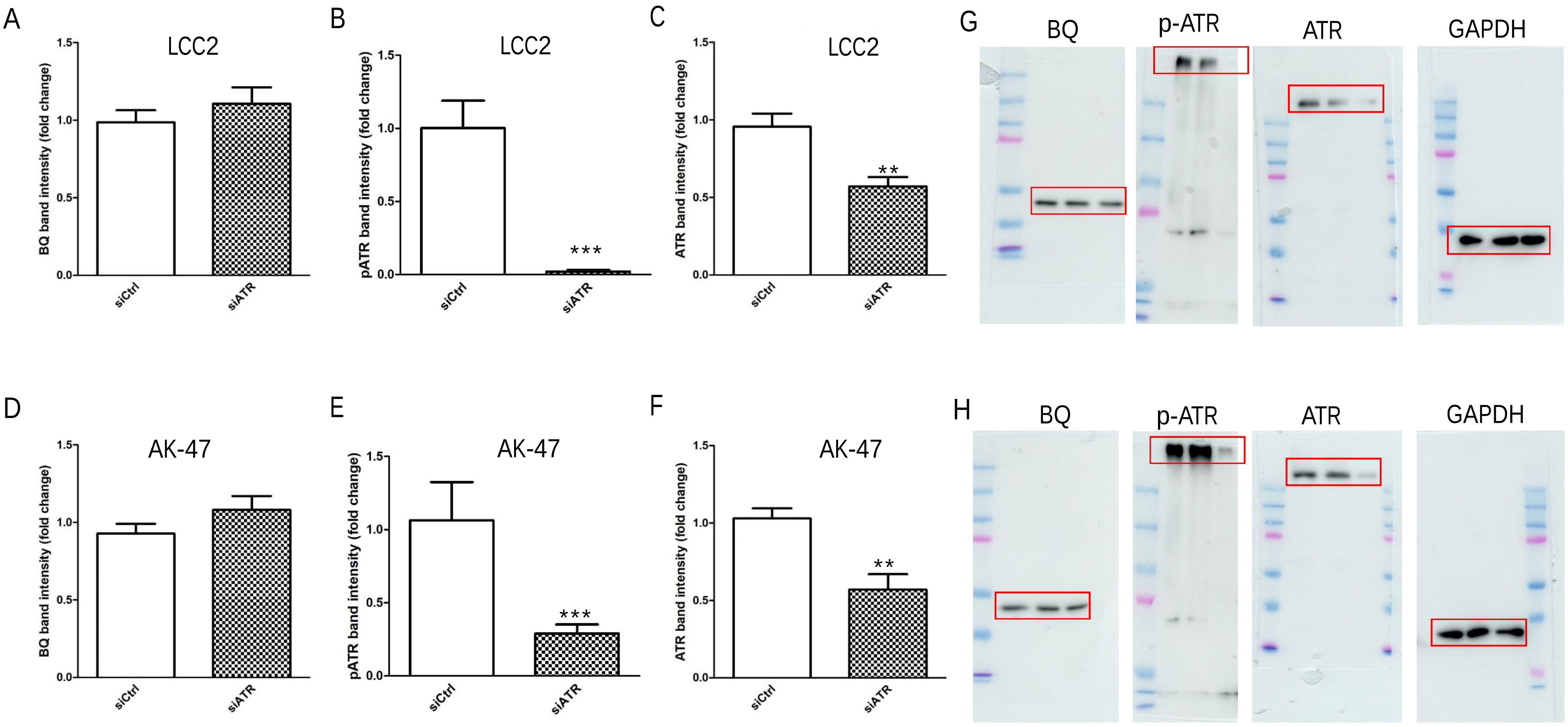
A
LCC2
BQ band intensity (fold change)
1.5
1.0
0.5
0.0
siCtrl
siATR
B
LCC2
pATR band intensity (fold change)
***
C
LCC2
ATR band intensity (fold change)
**
G
BQ
p-ATR
ATR
GAPDH
D
AK-47
E
AK-47
F
AK-47
H

Figure S8 Quantification of A) BQ, B) p-ATR (Ser1989) and C) ATR band intensity of the LCC2 panel in Figure 2C. Quantification of D) BQ, E) p-ATR (Ser1989) and F) ATR band intensity of the AK-47 panel in Figure 2C. G) Representative raw blots for the LCC2 panel in Figure 2C. H) Representative raw blots for the AK-47 panel in Figure 2C. Results were shown as mean ± SD from three independent experiments. Students' *t*-test was employed to compare the statistical significance between groups. ** and *** represent $p < 0.01$ and $p < 0.001$, respectively.

Figure S9

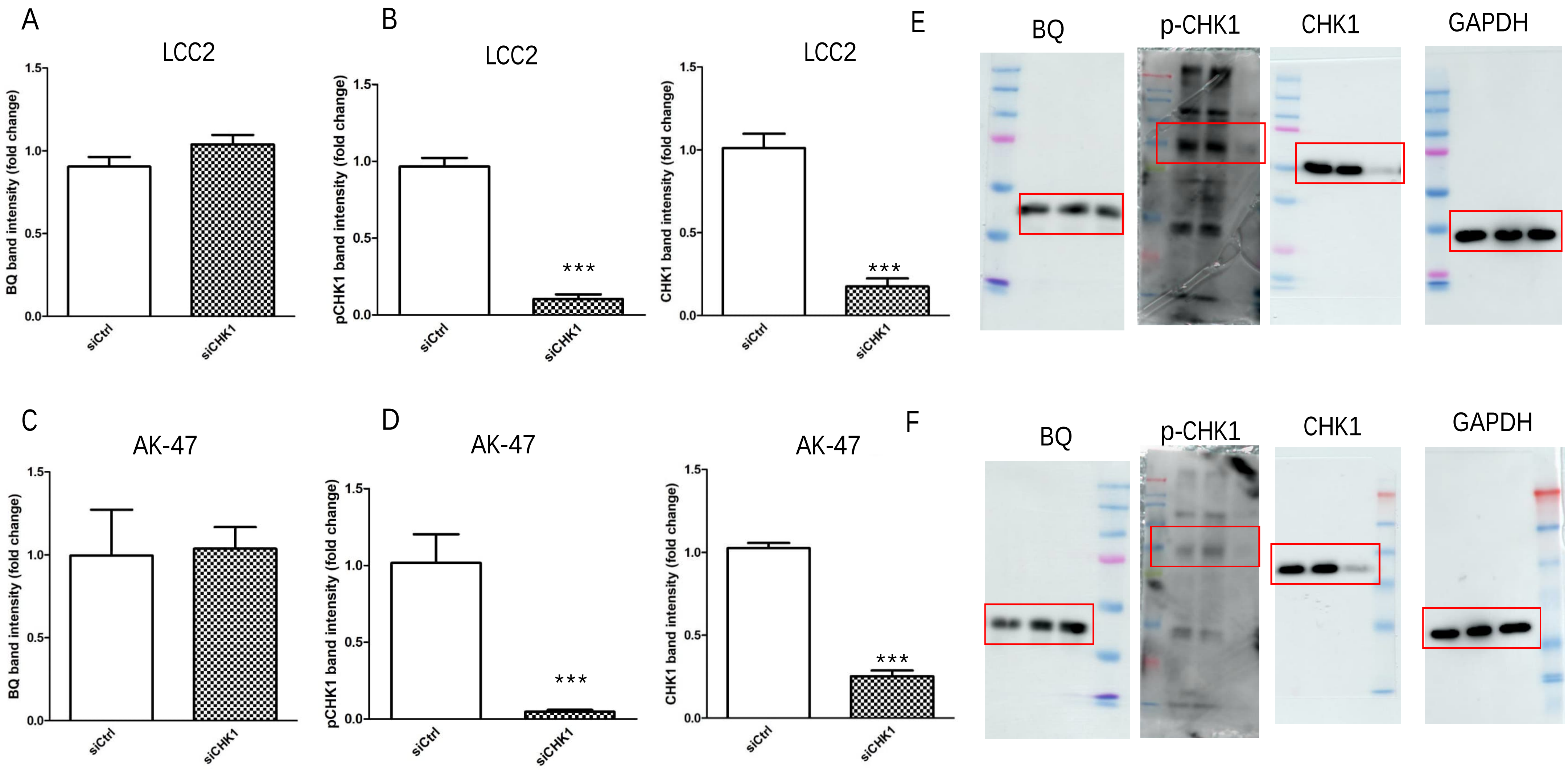

A
LCC2
BQ band intensity (fold change)
1.5
1.0
0.5
0.0
siCtrl
siCHK1
B
LCC2
pCHK1 band intensity (fold change)
***
CHK1 band intensity (fold change)
E
BQ
p-CHK1
CHK1
GAPDH
C
AK-47
D
F

Figure S9 Quantification of A) BQ, B) p-CHK1 (Ser345) and C) CHK1 band intensity of the LCC2 panel in Figure 2D. Quantification of D) BQ, E) p-CHK1 (Ser345) and F) CHK1 CHK1 band intensity of the AK-47 panel in Figure 2D. G) Representative raw blots for the LCC2 panel in Figure 2D. H) Representative raw blots for the AK-47 panel in Figure 2D. Results were shown as mean ± SD from three independent experiments. Students' t-test was employed to compare the statistical significance between groups. *** represents $p < 0.001$.

Figure S10

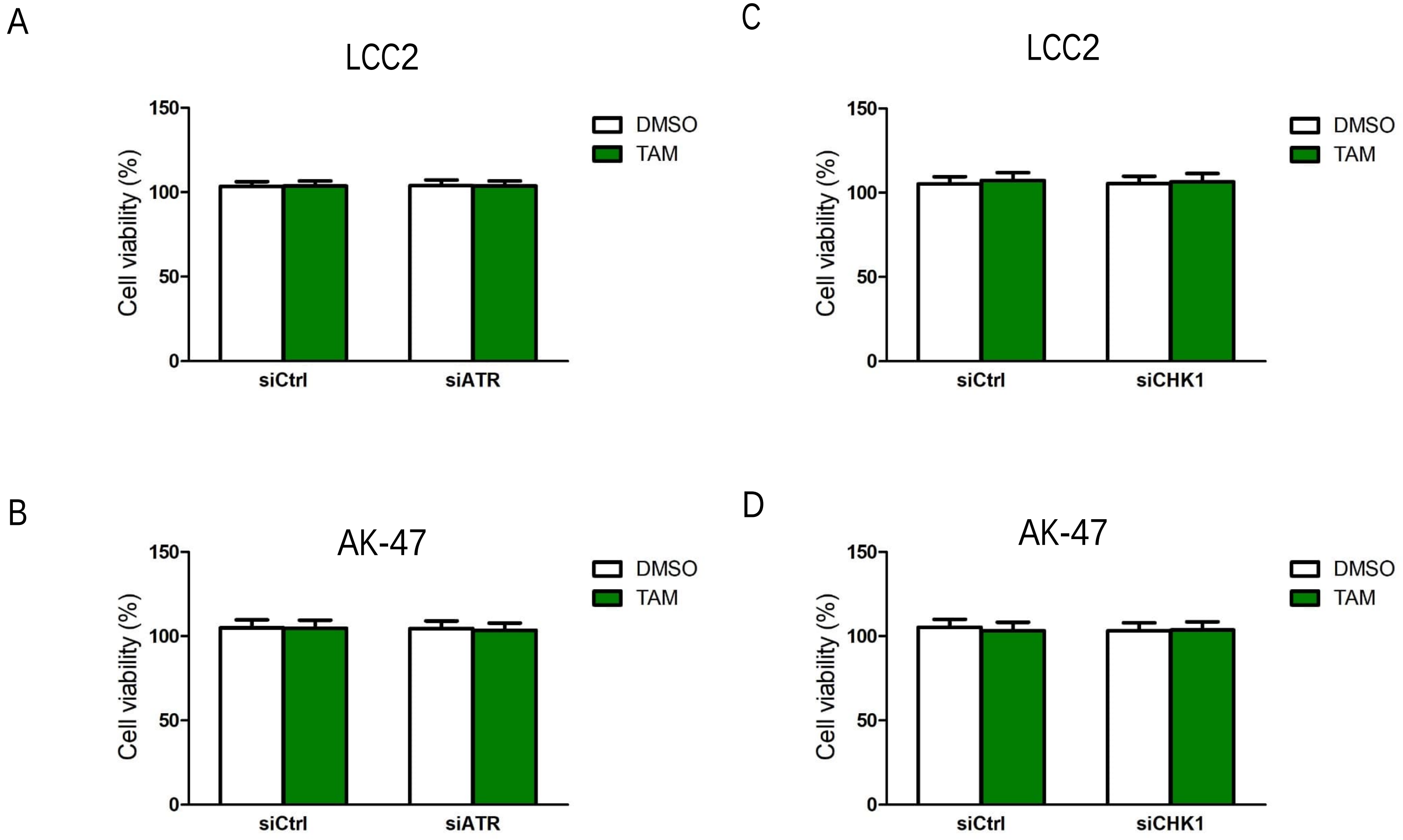
A
LCC2
Cell viability (%)
150
100
50
0
siCtrl
siATR
DMSO
TAM
B
AK-47
Cell viability (%)
150
100
50
0
siCtrl
siATR
DMSO
TAM
C
LCC2
Cell viability (%)
150
100
50
0
siCtrl
siCHK1
DMSO
TAM
D
AK-47
Cell viability (%)
150
100
50
0
siCtrl
siCHK1
DMSO
TAM

Figure S10 The effect of ATR knockdown in A) LCC2 and B) AK-47 on TAM response. The effect of CHK1 knockdown in C) LCC2 and D) AK-47 on TAM response. The cells were treated 20 nM of the siRNA and 4 μM of TAM for 96 hours. MTT assay was performed to determine cell viability. Results were shown as mean ± SD from six independent experiments. Students' t-test was employed to compare the statistical significance between TAM and DMSO groups.

Figure S11

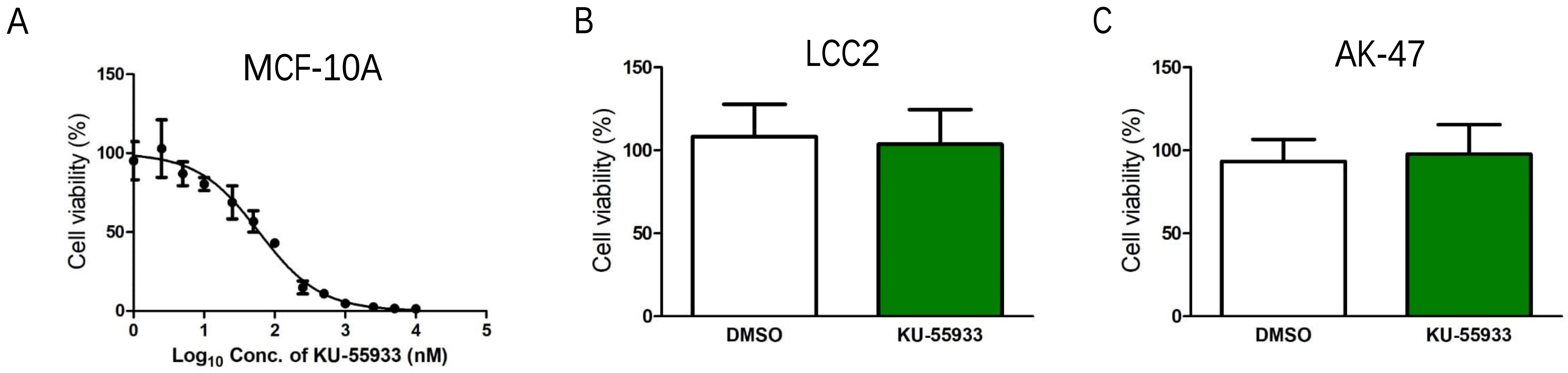
A
MCF-10A
Cell viability (%)
150
100
50
0
0
1
2
3
4
5
Log_{10} Conc. of KU-55933 (nM)
B
LCC2
Cell viability (%)
150
100
50
0
DMSO
KU-55933
C
AK-47
Cell viability (%)
150
100
50
0
DMSO
KU-55933

Figure S11 The effect of KU-55933 on A) MCF-10A. MCF-10A cells were treated with different concentrations of KU-55933 for 96 hours. MTT assay was performed. Results were shown as mean ± SD from four independent experiments. 5 nM was the maximum non-lethal dosage. The effect of KU-55933 on B) LCC2 and C) AK-47 on cell viability. The cells were treated with 5 nM of KU-55933 for 96 hours. MTT assay was performed. Results were shown as mean ± SD from four independent experiments. Students' *t*-test was employed to compare the statistical significance between KU-55933 and DMSO groups.

Figure S12

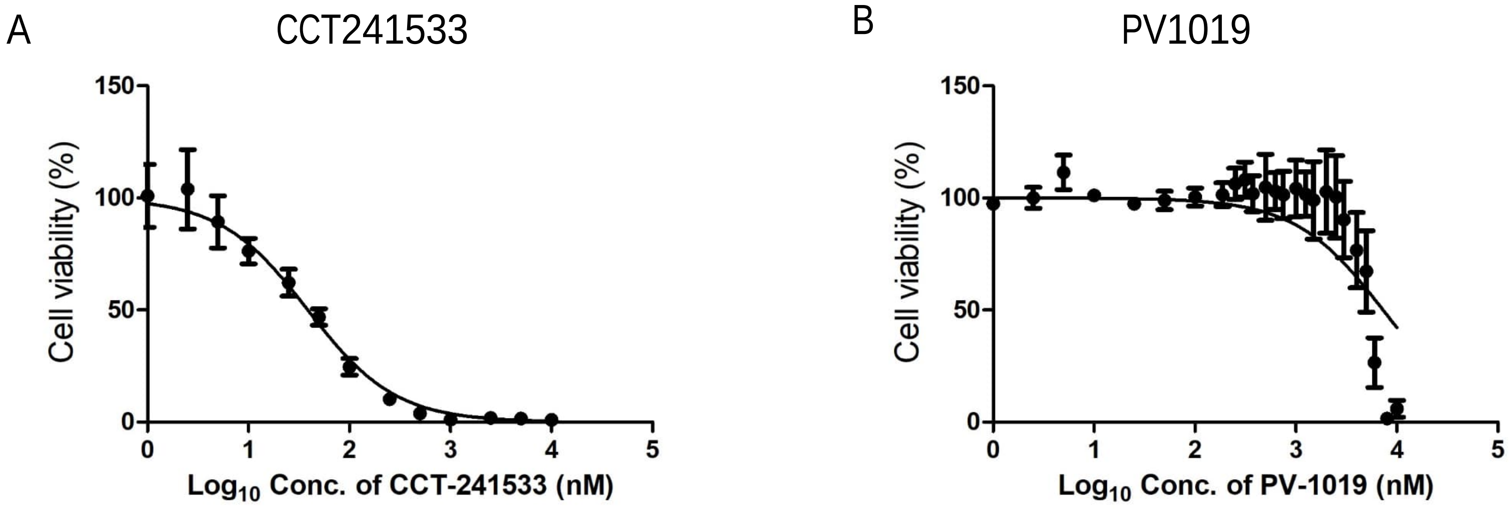
A
CCT241533
150
100
50
0
Cell viability (%)
0
1
2
3
4
5
Log10 Conc. of CCT-241533 (nM)
B
PV1019
150
100
50
0
Cell viability (%)
0
1
2
3
4
5
Log10 Conc. of PV-1019 (nM)

Figure S12 The effect of A) CCT-241533 and B) PV1019 on the cell viability of MCF-10A. The cells were treated with different concentrations of CCT241533 and PV1019 for 96 hours. MTT assay was performed. Results were shown as mean ± SD from four independent experiments. 10 nM of CCT241533 and 2 μM of PV1019 were the maximum non-lethal dosage.

Figure S13

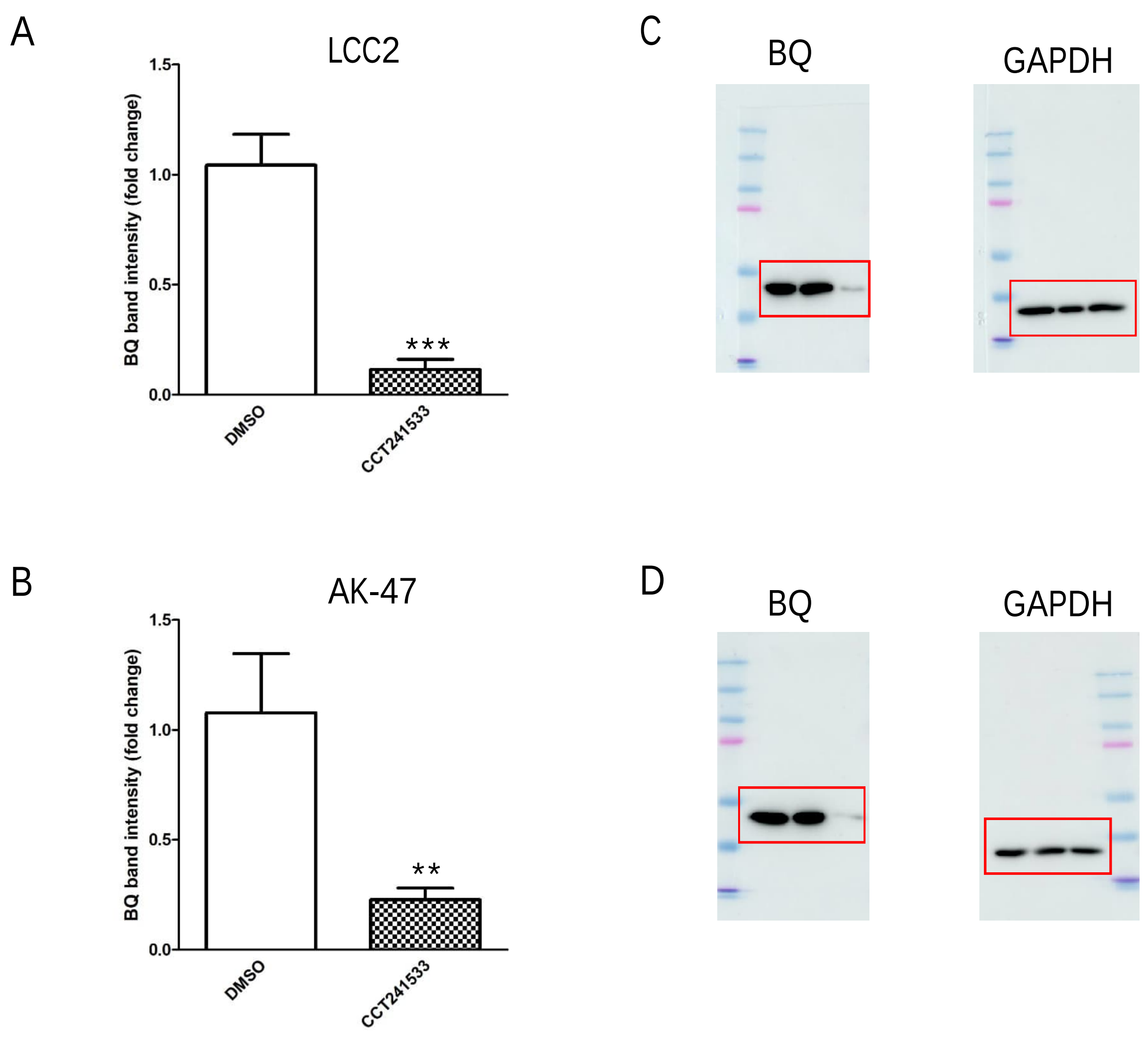
A
LCC2
1.5
1.0
0.5
0.0
BQ band intensity (fold change)
***
DMSO
CCT241533
B
AK-47
1.5
1.0
0.5
0.0
BQ band intensity (fold change)
**
DMSO
CCT241533
C
BQ
GAPDH
D
BQ
GAPDH

Figure S13 Quantification of BQ band intensity in A) LCC2 and B) AK-47 panel in Figure 5C. C) Representative raw blots for the LCC2 panel in Figure 5C. D) Representative raw blots for the AK-47 panel in Figure 5C. Results were shown as mean ± SD from three independent experiments. Results were shown as mean ± SD from three independent experiments. Students' t-test was employed to compare the statistical significance between groups. ** and *** represent $p < 0.01$ and $p < 0.001$, respectively.

Figure S14

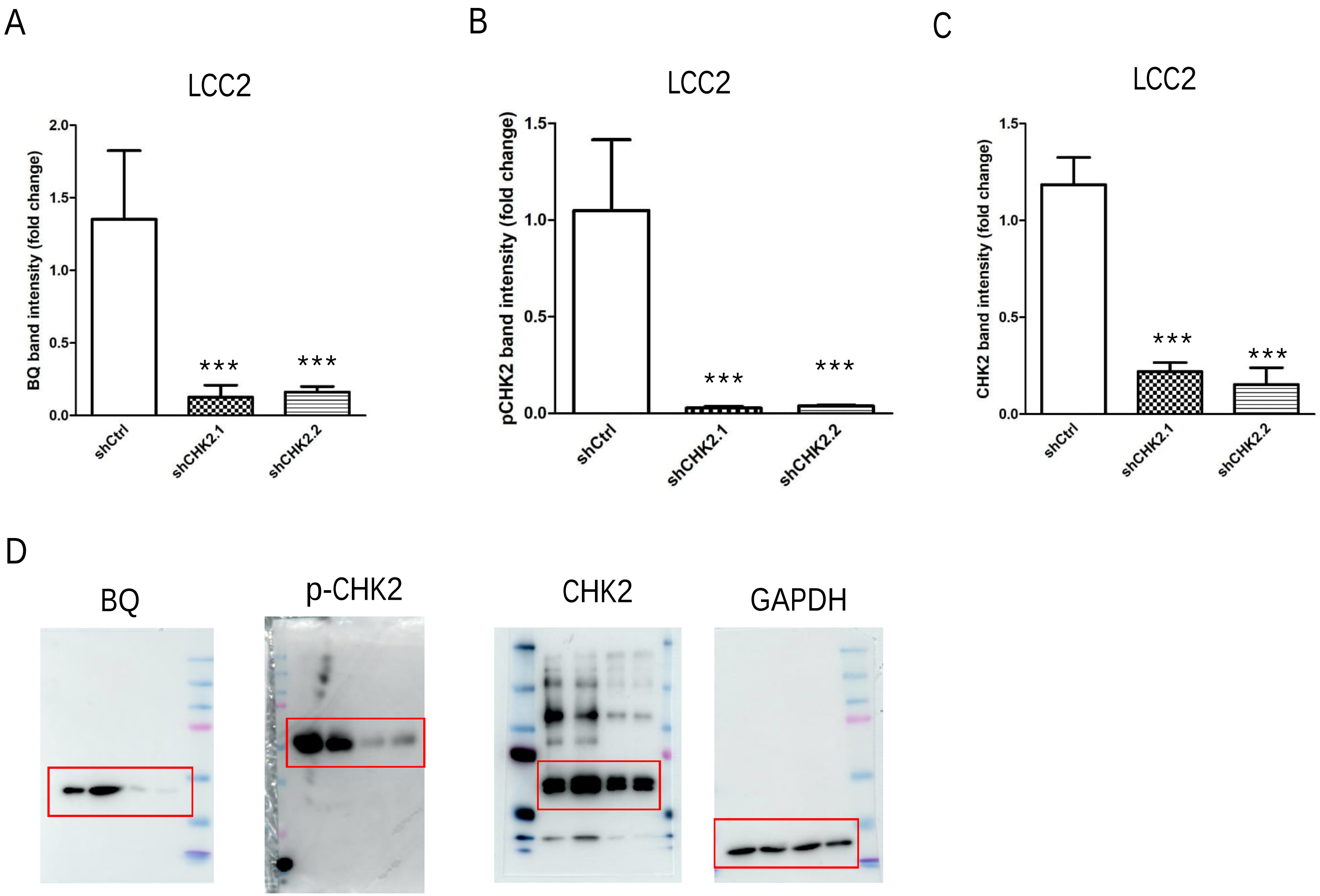
A
LCC2
BQ band intensity (fold change)
2.0
1.5
1.0
0.5
0.0
***
***
shCtrl
shCHK2.1
shCHK2.2
B
LCC2
pCHK2 band intensity (fold change)
1.5
1.0
0.5
0.0
***
***
shCtrl
shCHK2.1
shCHK2.2
C
LCC2
CHK2 band intensity (fold change)
1.5
1.0
0.5
0.0
***
***
shCtrl
shCHK2.1
shCHK2.2
D
BQ
p-CHK2
CHK2
GAPDH

Figure S14 Quantification of A) BQ B) p-CHK2 (Thr68) and C) CHK2 in Figure 5D. D) Representative raw blots for Figure 5D. Results were shown as mean ± SD from three independent experiments. Results were shown as mean ± SD from three independent experiments. One-way ANOVA with Bonferroni's post-test was employed to compare the statistical significance with shCtrl. *** represents $p < 0.001$.

Figure S15

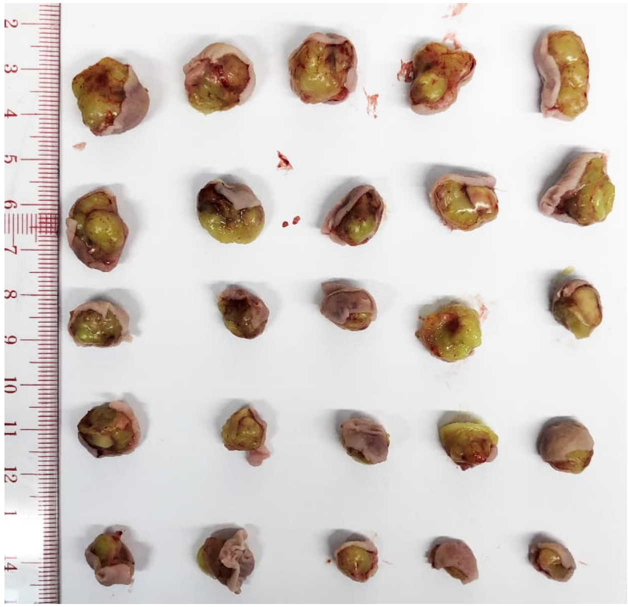

Saline

TAM

TAM + 1 mg/Kg CCT241533

TAM + 2.5 mg/Kg CCT241533

TAM + 5 mg/Kg CCT241533

Figure S15 CCT241533 could reverse TAM resistance in vivo. The image shows the tumours at the time of harvest in Figure 5F.

Figure S16

CHK2
IP:CHK2; IB CHK2

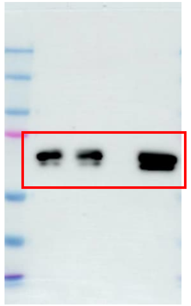

BQ
IP:CHK2; IB: BQ

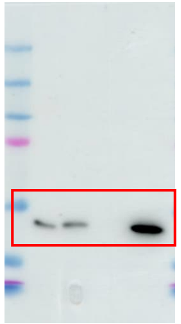

Figure S16 Representative raw blots for Figure 6A.

Figure S17

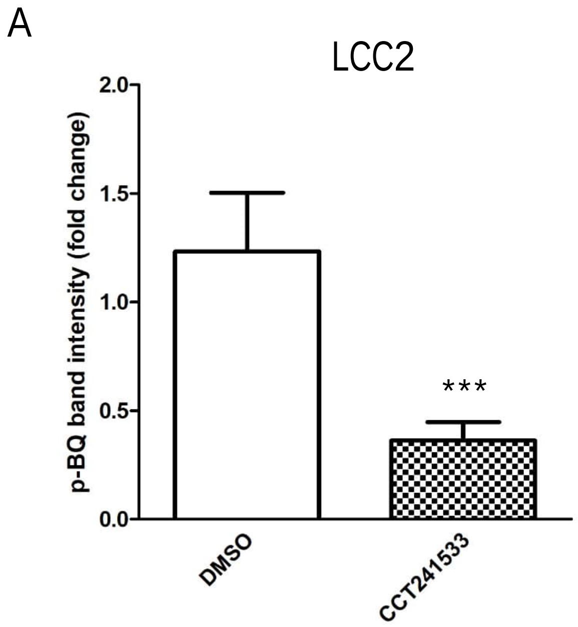
A
LCC2
p-BQ band intensity (fold change)
2.0
1.5
1.0
0.5
0.0
***
DMSO
CCT241533

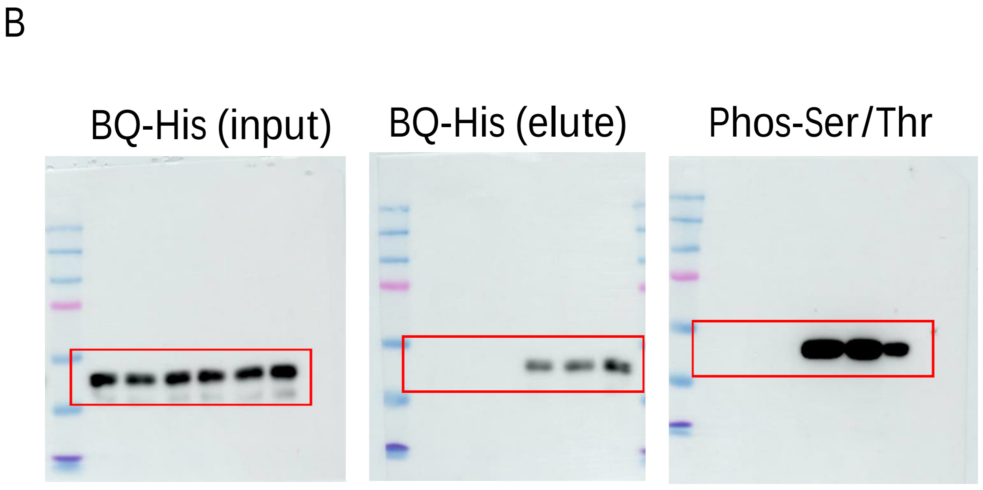
B
BQ-His (input)
BQ-His (elute)
Phos-Ser/Thr

Figure S17 A) Quantification of p-BQ in Figure 6B. B) Representative raw blots for Figure 6B. The signal in phos-Ser/Thr was relative to BQ elute. Fold change relative to untreated control was calculated. Results were shown as mean ± SD from three independent experiments. Results were shown as mean ± SD from three independent experiments. Students' t-test was employed to compare the statistical significance between groups. *** represents $p < 0.001$.

Figure S18

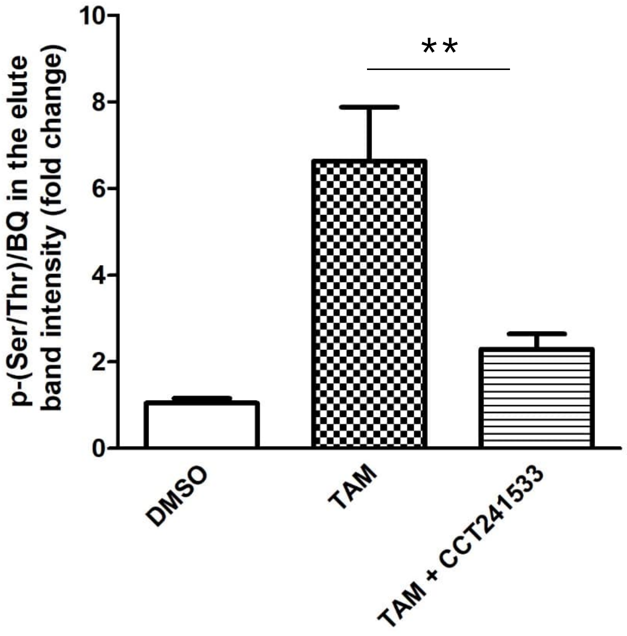
p-(Ser/Thr)/BQ in the elute band intensity (fold change)
10
8
6
4
2
0
**
DMSO
TAM
TAM + CCT241533

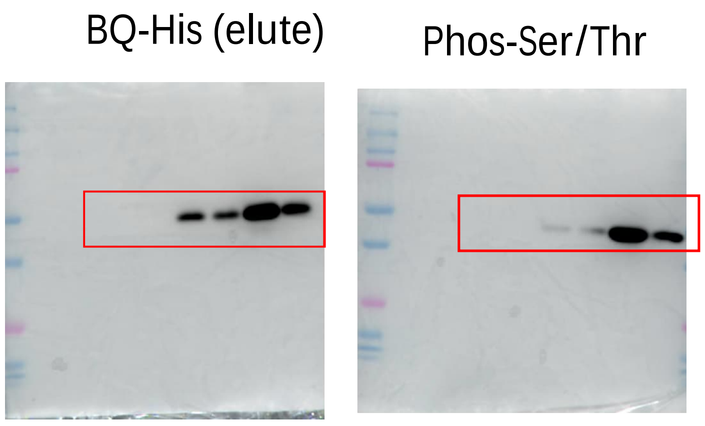
BQ-His (elute)
Phos-Ser/Thr

Figure S18 Quantification of p-BQ in Figure 6C. The signal in phos-Ser/Thr was relative to BQ elute. Fold change relative to untreated control was calculated. Results were shown as mean ± SD from three independent experiments. Results were shown as mean ± SD from three independent experiments. One-way ANOVA with Bonferroni's post-test was employed to compare the statistical significance between indicated groups. ** represents $p < 0.01$.

Figure S19

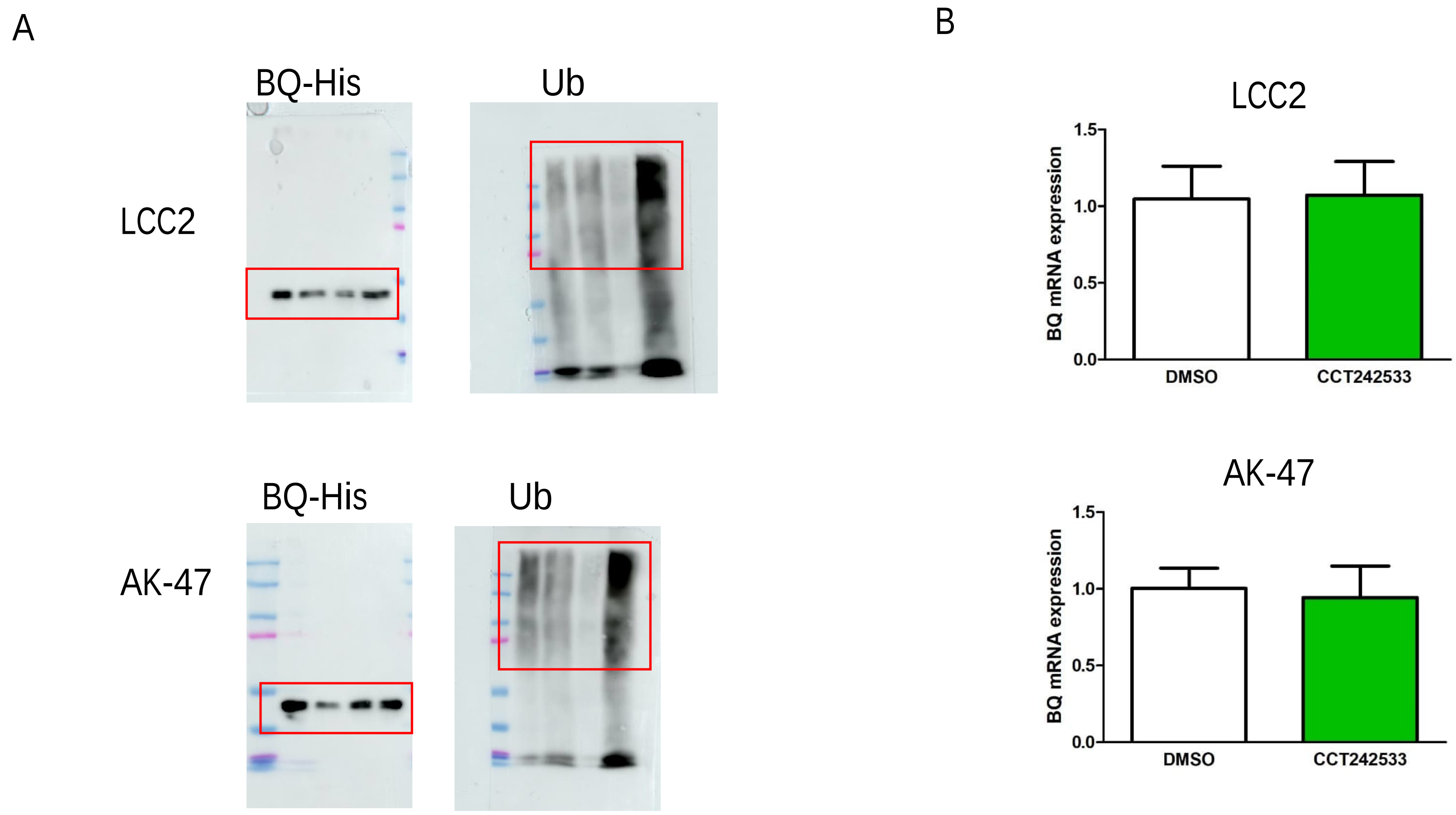
A
BQ-His
Ub
LCC2
BQ-His
Ub
AK-47
B
LCC2
1.5
1.0
0.5
0.0
BQ mRNA expression
DMSO
CCT242533
AK-47
1.5
1.0
0.5
0.0
BQ mRNA expression
DMSO
CCT242533

Figure S19 A) Representative raw blots for Figure 6D. B) The effect of CCT241533 on mRNA level of BQ in LCC2 and AK47 cells. qPCR was employed. Results were shown as mean ± SD from six independent experiments. Students' *t*-test was employed to compare the statistical significance between groups. There was no statistical significance between DMSO and CCT241533 groups.
